# Phytochemical Composition and Antimicrobial Activity of *Corydalis solida* and *Pseudofumaria lutea*

**DOI:** 10.3390/molecules25163591

**Published:** 2020-08-07

**Authors:** Sylwia Zielińska, Magdalena Dziągwa-Becker, Ewelina Piątczak, Anna Jezierska-Domaradzka, Malwina Brożyna, Adam Junka, Mariusz Kucharski, Serhat Sezai Çiçek, Christian Zidorn, Adam Matkowski

**Affiliations:** 1 Department of Pharmaceutical Biology, Wroclaw Medical University, Borowska 211, 50-556 Wrocław, Poland; anna.jezierska-domaradzka@umed.wroc.pl (A.J.-D.); pharmaceutical.biology@wp.eu (A.M.); 2 Department of Weed Science and Tillage Systems, Institute of Soil Science and Plant, Cultivation State Research Institute, Orzechowa 61, 50-540 Wrocław, Poland; m.dziagwa@iung.wroclaw.pl (M.D.-B.); m.kucharski@iung.wroclaw.pl (M.K.); 3 Department of Biology and Pharmaceutical Botany, Medical University of Łódź, Muszyńskiego 1, 90-151 Łódź, Poland; ewelina.piatczak@umed.lodz.pl; 4 Laboratory of Experimental Cultivation, Botanical Garden of Medicinal Plants, Wroclaw Medical University, Al. Jana Kochanowskiego 14, 50-556 Wroclaw, Poland; 5 Pharmaceutical Microbiology and Parasitology, Wroclaw Medical University, Borowska 211a, 50-556 Wroclaw, Poland; malwinabrozyna@gmail.com (M.B.); feliks.junka@gmail.com (A.J.); 6 Department of Pharmaceutical Biology, Kiel University, Gutenbergstraße 76, 24118 Kiel, Germany; scicek@pharmazie.uni-kiel.de (S.S.Ç.); czidorn@pharmazie.uni-kiel.de (C.Z.)

**Keywords:** *Corydalis solida*, *Pseudofumaria lutea*, protopine, protoberberine derivatives, quercetin

## Abstract

*Corydalis* and *Pseudofumaria* are two closely related genera from the *Papaveraceae* subfamily *Fumarioideae* with *Corydalis solida* (*C. solida*) and *Pseudofumaria lutea* (*P. lutea*) as two representative species. Phytochemical analysis revealed significant differences in the quality and quantity of isoquinoline alkaloids, phenolic compounds and non-phenolic carboxylic acids between aerial and underground parts of both species. Using the Liquid chromatography-electrospray ionization-tandem mass spectrometry (LC-ESI-MS/MS) technique, 21 compounds were identified: five protoberberine derivatives, three protopine derivatives, four phenanthridine derivatives, as well as three carboxylic acids, two hydroxycinnamic acids, one chlorogenic acid, one phenolic aldehyde, and two flavonoids. Moroever, significant differences in the content of individual compounds were observed between the two studied species. The phytochemical profile of *C. solida* showed a higher variety of compounds that were present in lower amounts, whereas *P. lutea* extracts contained fewer compounds but in larger quantities. Protopine was one of the most abundant constituents in *C. solida* (440–1125 µg/g d.w.) and in *P. lutea* (1036–1934 µg/g d.w.). Moreover, considerable amounts of coptisine (1526 µg/g) and quercetin (3247 µg/g) were detected in the aerial parts of *P. lutea*. Extracts from aerial and underground parts of both species were also examined for the antimicrobial potential against *S. aureus*, *P. aeruginosa* and *C. albicans*. *P. lutea* herb extract was the most effective (MIC at 0.39 mg/L) against all three pathogens.

## 1. Introduction

*Corydalis* DC. is the largest genus in the Fumarioideae subfamily belonging to the Papaveraceae family [1]. It has over 400 species varying in terms of life forms, such as rhizome perennials, early spring geophytes, therophytes and perennial climbing plants [2]. A small genus of *Pseudofumaria* Medik. was separated from the genus *Corydalis*, based on morphological traits only, e.g., the pistil, which is deciduous and translucent in *Pseudofumaria* and persistent and green in *Corydalis* [2,3]. *Pseudofumaria* comprises only two species: *Pseudofumaria lutea* (L.) Borkh (syn. *Corydalis lutea* (L.) DC.) and *P. alba* (Mill.) Lidén (syn. *Corydalis alba* (Mill.) Mansf) [3,4,5].

In *Corydalis*, several species have been described during the recent few decades rendering a complex and not fully understood taxonomic relationship [6]. However, only some of the species and infraspecific taxa were subjected to phytochemical analysis with over 50 isoquinoline alkaloids listed [7,8,9,10]. Yet, the phytochemical relationship to the other taxa within the Fumarioideae subfamily is not fully documented. Therefore, we have chosen two typical and most representative examples of each genus, i.e., *Corydalis solida* (L.) Clairv and *Pseudofumaria lutea* to compare their phytochemical profiles.

*C. solida* is found almost all over Europe, except in the very Northern and Western regions. It is found in the lowlands, foothills and in the low mountain regions as an early spring geophyte associated with European oak-hornbeam forests. It is forming corms, forming small underground bulbs or bulbo-tubers, which unlike a similar species of *C. cava*, are full inside. From the corms grows a single raised stem with two 2- to 3-pinnate leaves and the top racemose inflorescence of purple, monosymmetrical flowers [11]. *C. solida* is also considered a type species for the genus, originally described as *solida* subspecies of a basionym *Fumaria bulbosa* [12,13].

*P. lutea* grows in Italian and Swiss Alps on shady limestone rocks and screes at an altitude of 500-1700 m a.s.l. As an ornamental plant, it has spread almost all over Europe, and in many countries, including Poland, acquired the status of an established anthropophyte [3,14,15]. The place of its occurrence are mostly rocky places and stone walls, always on calcareous soils. *P. lutea* is a rhizome perennial. Stems is branched, with many 2- to 3-pinnate leaves. The plant produces yellow flowers gathered in racemes. The flowering period is May to October [2].

Data on the traditional or phytomedicinal use of both plants are scarce, which is probably related to the alleged toxicity. *C. solida* was sometimes used as a calming plant, pain reliever, lowering blood pressure. Starch-rich tubers were cooked and eaten by Slavic peoples and Tatars [16,17]. In Serbia, tubers were used as a sedative, against bleeding, scurvy and worms [18]. Even less data is available for *P. lutea*. Only one source reports the use of the herb as a gout treatment [19].

Little data on the phytochemical characterization of these two species prompted us to perform comparative analyses. In aerial and underground parts of the plants cultivated in the same location, we analyzed not only alkaloids but also carboxylic acids and various phenolic compounds. The phytochemical analysis was performed using Liquid chromatography-electrospray ionisation-tandem mass spectrometry (LC-ESI-MS/MS) technique.

Insufficient available data on the phytochemistry of these two species prompted us to perform comparative analysis using LC-MS. In the aerial and underground parts of the plants cultivated in the same location, we analyzed not only alkaloids but also carboxylic acids and various phenolic compounds. The phytochemical analyses were set for a better insight into the relationships between the species that were apparently diversified enough, to separate the *Pseudofumaria* genus from the *Corydalis*. Changes in the taxonomic position of the species were based on morphological features only, therefore phytochemical characteristics may provide additional valuable information about the closely related and yet different taxa. Moreover, the antimicrobial properties against selected pathogenic bacteria and fungi (*P. aeruginosa*, *S. aureus*, *C. albicans*) as expected from the high alkaloid content, provide a foundation for phytotherapeutic potential of these underutilized herbs.

## 2. Results

### 2.1. Qualitative Analysis

Phytochemical analysis of aerial and underground parts of *C. solida* and *P.lutea* revealed two major groups of metabolites in the extracts, benzophenanthridine alkaloids and polyphenolic compounds. A total of 21 compounds were detected—twelve in negative and nine in positive electrospray ionization mode. Two protoberberine derivatives: coptisine and berberine, two protopine derivatives: protopine and allocryptopine, and three phenanthridine derivatives: sanguinarine, chelerythrine, and chelidonine, as well as five of their derivatives: a protopine derivative, a coptisine derivative, tetrahydrocoptisine, tetrahydroberberine and a chelidonine derivative were identified. Among the non-alkaloid compounds, there were: three carboxylic acids, two hydroxycinnamic acids, one quinic acid ester, one phenolic aldehyde and two flavonoids (Table 1 and Table 2).

The assignment of allocryptopine was based on the parent ion at *m/z* 369 and the product ions at *m/z* 352, 188, 290. Protopine showed the precursor ion at *m/z* 320, and a putative protopine derivative at *m/z* 354 with product ions at *m/z* 320, 260, 196. Coptisine gave parent ion at *m/z* 320. Tetrahydrocoptisine and a putative coptisine derivative showed parent ions at *m/z* 324. The assignment of berberine was based on the parent ion at *m/z* 336. Its derivative—tetrahydroberberine—showed the parent ion at *m/z* 340 and product ions at *m/z* 176, 149. The most abundant precursor ions at *m/z* 332 and 348 were assigned for sanguinarine and chelerythrine, respectively. Chelidonine exhibited the parent ion at *m/z* 370 and product ions at *m/z* 356 and 339 (Table 1 and Table 2).

Malic acid presence was based on the parent ion at *m/z* 133, trans-aconitic acid at *m/z* 173, and quinic acid at *m/z* 191. The assignment of *p*-coumaric acid and *trans*-caffeic acid was based on the parent ions at *m/z* 163 and 179, respectively. Chlorogenic acid showed the parent ion at *m/z* 353. The assignment of two flavonoids—rutin and quercetin was based on the presence of parent ions at *m/z* 609 and 301, respectively. The identification of vanillin was based on the parent ion at *m/z* 151 and quinine sulfate at *m/z* 747 (Table 1 and Table 2).

### 2.2. Quantitative Analysis

**Figure 1:**
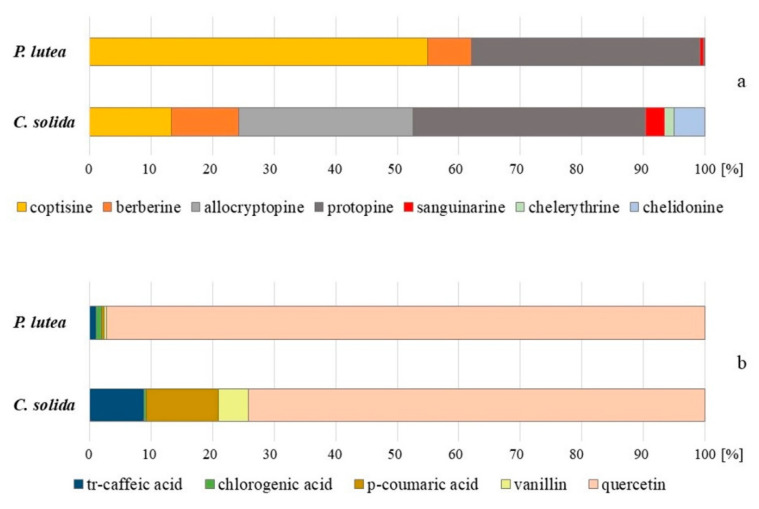
Isoquinoline alkaloids (**a**) and phenolic compounds (**b**) proportions in aerial parts of *P. lutea* and *C. solida*.

**Figure 2:**
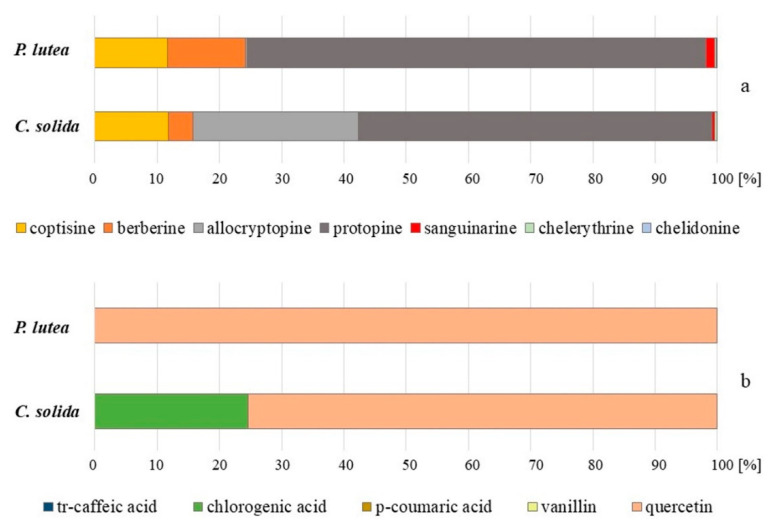
Isoquinoline alkaloids (**a**) and phenolic compounds (**b**) proportions in underground parts of *P. lutea* and *C. solida*.

The quantities of most of the detected compounds varied significantly between the species and organs and the proportions between each compound made up markedly different profiles (Figure 1 and Figure 2). Protopine and its derivative were present in aerial and underground parts of both studied species. The content of protopine varied between 440 and 1036 µg/g of dry weight (d.w.) in the aerial parts, and between 1125 and 1934 µg/g d.w. in underground parts of *C. solida* and *P. lutea*. Allocryptopine was present in significant concentrations in the herb and corms of *C. solida* (328, 516 µg/g d.w., respectively), and in low amounts (6 µg/g d.w.) in the herb and roots of *P. lutea* (Table 1 and Table 2). All five protoberberine derivatives were present in aerial and underground parts of the studied species. The coptisine content varied in the range of 307–1526 µg/g in *P. lutea* and 154–233 µg/g in *C. solida*. Berberine amounts varied between 197 and 326 µg/g in *P. lutea*, and between 128 and 78 µg/g in *C. solida*. The amounts of phenanthridine derivatives, such as sanguinarine, chelethrine and chelidonine in both species ranged from 1 to 36 µg/g d.w. (Table 1 and Table 2).

Ten different polyphenolic compounds were detected in aerial parts and seven in underground parts of *C. solida* and *P. lutea* (Table 1 and Table 2). *Trans*-caffeic acid was present only in aerial parts (21–32 µg/g d.w.), and so were *p*-coumaric acid (16–28 µg/g d.w.), vanillin (11–13 µg/g d.w.) and rutin (LOQ). Chlorogenic acid (1–32 µg/g d.w.) and quercetin (19–3247 µg/g d.w.) were found in aerial and underground parts of both species (Table 1 and Table 2).

### 2.3. MIC Evaluation

To show potential biological applicability of gained extracts (from both aerial and underground parts of both species), we have performed microbiological tests to assess their usefulness in eradication of common nosocomial pathogens (*P. aeruginosa*, *S. aureus*, *C. albicans*).

Extracts of *C. solida* and *P. lutea* herbs and underground parts were examined for their antimicrobial potential in microtiter-plate based assay against the microbes in a suspension. All of the extracts exhibited strong antimicrobial activity against Gram-positive, Gram-negative bacteria and *C. albicans* yeasts. *P. lutea* herb extract was the most effective (MIC at 0.39 mg/L) against all of the three pathogens (Table 3). Root extract of the species as well as both extracts of *C. solida* exhibited weaker activity against *S. aureus* (MIC at 1.56 mg/L) than against other tested strains (MIC at 0.39 mg/L) (Table 3).

## 3. Discussion

The chromatographic analysis revealed diverse phytochemical profiles of *C. solida* and *P. lutea*. Isoquinoline alkaloids and the non-alkaloid compounds were found in aerial and underground parts of both studied species.

Herb and roots of *P. lutea* contained twice as much protopine as *C. solida*. Moreover, there was almost ten times more coptisine and almost twenty times more quercetin in the aerial parts of *P. lutea* than in those of *C. solida*. Additionally, *P. lutea* roots contained three times more berberine and sanguinarine than corms of *C. solida* (Table 1 and Table 2, Figure 1 and Figure 2). Protopine and protoberberine derivatives were previously found in *P. lutea* along with several other groups such as aporphine and two narceine derivatives. The latter two were soon recognized as artifacts [9,20].

In our research, the amounts of berberine in the herb and roots of *P. lutea* reached nearly 197 and 326 µg/g d.w., but its presence has not always been found in the previous studies on this species. Preininger et al. in 1978 [10] isolated fourteen different alkaloids from *P. lutea* among which two protoberberine derivatives—corysamine and palmatine. The identity of the compounds was confirmed by comparing their UV and IR spectra with the reference substances. The authors did not find berberine in their plant material, but they did not specify which plant organs were used. Moreover, no phenanthridine derivatives were detected.

In turn, the herb and corms of *C. solida* were from several dozen to several hundred times richer in allocryptopine than *P. lutea* (Table 1 and Table 2, Figure 1 and Figure 2). Additonally, *C. solida* herb contained several times more phenanthridine derivatives such as chelidonine, chelerythrine and sanguinarine than the other species (Table 1, Figure 1). Sanguinarine was previously detected in the corms of *C. solida* by Temizer et al. in 1992 [21]. The authors also found three protoberberine derivatives such as berberine, ophiocarpine and scoulerine, and one alkaloid from another chemical group—protopine. Tubers of *C. solida* examined by Sturm et al. in 2007 [22] also contained two protoberberine derivatives (corydine, palmatine), and one aporphine derivative bulbocapnine. No protopine and phenanthridine type alkaloids were detected in their plant material [22]. In turn, from the extract of whole *C. solida* plants, a spirobenzylisoquinoline alkaloid named corysolidine was isolated in 1986 by Rahimizadeh et al. [23]. On the other hand, Kilic et al. in 2019 [24], similarly to our results, found large proportions of protopine in the corms of *C. solida* ssp. *incisa.* The plant material was collected in Turkey, and five protopine type alkaloids and eight protoberberine type were detected in the extracts. The authors used an advanced LC-QTOF-MS technique for the compounds identification, nevertheless it is difficult to compare their results with ours, because they examined the subspecies of *C. solida*.

Yet in other studies, on another taxon—*Corydalis solida* subsp. *tauricola*—a total of 23 alkaloids were isolated from aerial parts, among which no protopine was identified. Twenty-one of them were previously identified (allocryptopine, berberine, ophiocarpine, scoulerine, sinactine, corydalidzine, dehydrocorydaline, sanguinarine, norsanguinarine, bulbocapnine, isoboldine, reticuline, α-hydrastine, bicuculline, ochotensine, sibiricine, oxocularine, oxosarcocapnidine, fumariline, cularine), and two were new (taurine, tauricoline) [25]. All of these structures can be found in PubChem or ChemSpider databases but tauricoline—this alklaoid has been only mentioned in the study of Sener et al. (1990) [25]. Some other authors used extracts containing alkaloids but they presented only the TLC chromatogram (visualized in UV_365nm_) of *C. solida* ssp. *slivenensis* and *C. solida* ssp. *laxa* herbs and tubers [26]. The authors presented their results only without the use of reference substances. *Corydalis solida* ssp. *laxa* from Steninge close to Uppsala (Sweden) is probably a hybrid between *C. solida and C.pumila* as herbarium specimens from 1948 combine the features of both species. It should be noted that *C. solida* observed on Aland and the Uppland coast, on the eastern coast of Sweden, was also called “laxa”, but it presents morphologically diversified characteristics such as ovaries that smoothly attenuate into a short style, lower petals free of gibbosity, and broad leaf lobes [27].

Trying to capture similarity or indicate differences between these taxa, it is also worth paying attention to the phytochemical profile of these species (Figure 1 and Figure 2). Unfortunately, the published data for various taxa, additionally collected from different locations are insufficient. Regarding phytochemical data for *C. pumila*, one published research study can be found. It has been presented that in corms of *C. pumila*, several alkaloids such as bulbocapine, corydine, corydaline, palmatine, tetrahydropalmatine were detected [22], but no more detailed phytochemical characteristics are available in the literature. Our results showed that, except quercetin, both plants contained relatively small and comparable amounts of non-phenolic carboxylic acids (malic, trans-aconitic, quinic), hydroxycinnamic acids (*trans*-caffeic acid and *p*-coumaric acid), chlorogenic acid, and vanillin in aerial parts (Table 1). The underground parts were even poorer in these metabolites (Table 2, Figure 1 and Figure 2). Conversely, malic acid was previously detected in considerable amounts of 3.9 mg/g in the herb aqueous extracts of *P. lutea*, and so were (-)-caffeoylmalic acid, *p*-coumaroylmalic acid, feruloyl-malic acid, caffeic acid, *p*-coumaric acid, ferulic acid, sinapic acid [28].

Since the observations of the morphology and taxonomy of species within the genus *Corydalis* show many complexities, and there are very few data on the phytochemistry of these plants, we believe that there is a need for comprehensive phytochemical studies of representatives of the current *Corydalinae* subtribe. Based on our research, further detailed analyses would allow us to determine the proportions between individual compounds, especially these of alkaloid and polyphenol groups contained in many other *Corydalis* species, and thus select the most valuable raw material. This would be justified because of the wide spectrum of biological activities of both isoquinoline alkaloids and polyphenolic compounds confirmed in the literature, especially antimicrobial [29,30,31,32,33,34,35].

From all tested samples, the herb extract of *P. lutea* was the most effective against all three pathogens (MIC at 0.39 mg/L, Table 3). It contained large proportions of coptisine, berberine, protopine and quercetin (Table 1). In our earlier studies, individually tested protopine and coptisine, presented antibacterial properties against *S. aureus* at a concentration of less than 50 mg/L (73% CFU reduction), while berberine was less effective (MIC at 125 mg/L). Additionally, coptisine showed antibacterial activity against *P. aeruginosa* and *C. albicans*, similar to allocryptopine (MIC at > 50–125 mg/L) [35]. Protopine and protoberberine derivatives such as coptisine and berberine have several pharmacological activities. Beside antimicrobial activity, analgesic, anti-inflammatory, anticancer, antithrombotic, hypoglycaemic, hypolipidaemic, hepatoprotective, and neuroprotective properties were also reported [36,37,38,39]. Apart from protoberberine alkaloids, several benzophenanthridine derivatives, such as chelidonine, chelerythrine and sanguinarine were also present in extracts of *C. solida* and *P. lutea*. Although these substances were detected in relatively small amounts (from dozen or so to several dozen µg/g d.w., Table 1 and Table 2), they may cause the reinforcement of the antimicrobial properties of extracts. Furthermore, the contribution of polyphenolic constituents in the plant extract, should not be neglected. This class of compounds may be strongly involved in the microbes eradication [40]. In one of the most recent studies on polyphenolic compounds contained in extracts of *Anthemis praecox* aerial parts, quercetin rich extracts presented antimicrobial activity, although stronger against Gram-positive than Gram-negative bacteria [41]. It should be noted that a general trend in antimicrobial activity presented in Table 3 indicates higher efficiency of tested extracts against *P. aeruginosa* and *C. albicans* than against *S. aureus*. In the studies of Orhan et al. (2006) [42], a large number of alkaloids that were isolated from several *Corydalis* and *Fumaria* species, were also more active against Gram-negative than Gram-positive bacteria. *P. aeruginosa* is closely related with *Pseudomonas putida*, a microbial cohabitant of *C. solida* and *P. lutea* environment and growth-promoting rhizobacteria. Several deadly plant pathogen strains belong to *Pseudomonas syringae.* Yeast-like fungi, to whom *C. albicans* belongs to, can colonize, co-exist or cause diseases in a vast variety of plant species. In turn, *S. aureus*, is a highly-specialized opportunistic pathogen of animal and men. One may thus assume that observed higher potential of analyzed plants to eradicate *C. albicans* and *P. aeruginosa* than *S. aureus* may be related to the ability of analyzed plants to combat microorganisms which exist in plants’ habitat in order to avoid the negative impact of microbial overgrowth [43,44,45,46]. This hypothesis, along with the presented data on the higher antimicrobial efficacy of plant extracts versus individually tested alkaloids, indicates the necessity of further detailed phytochemical and bioactivity investigations. The complex analysis would allow to set down new plant-derived products of desired medicinal properties.

## 4. Material and Methods

### 4.1. Plant Material

Whole plants of *C. solida* and *P. lutea* were collected from Botanical Garden of Maria Curie-Skłodowska University in Lublin on 20th of April 2018. *C. solida* was replanted from Stone pit of Kazimierz Dolny, Poland in 1976, *P. lutea* was replanted from the garden of a private person from Świdnik, Poland in 2006, and from that time both species are in the collection of Botanical Garden of Maria Curie-Skłodowska University in Lublin (Geographical location: 51°6′ N, 22°30′ E, 200 m a.s.l.), under the codes: 1910P, 4223A, respectively. Plants grew on loess soils without additional treatments such as fertilization. *C. solida* grew in a shadowed place, *P. lutea* grew in an exposed, sunny location. The average temperature in April 2018 was 14.2 °C and precipitation was 36.5 mm (data from the official report of the Meteorological Observatory of the Meteorology and Climatology Department, the Maria Curie-Skłodowska University in Lublin). Continental influences with large amplitudes of annual temperatures, a long summer and a long cool winter predominate in the Lublin Upland. During the years 1951–2010 average annual perennial temperature was +8.3 °C, and average annual multi-annual precipitation was 550.6 mm.

Plants were collected, dried in a heated herbal drier at 25 °C for 72 h and separated into aerial and underground parts. In case of *C. solida* there were corms, and in case of *P. lutea*–roots.

#### Plant Material Extraction

Ten individual plants of each species were used for extraction. Three independent experimental repetitions were performed followed by two analytical repetitions. Dried plant material was ground to powder using mortar and pestle and extracted with 80% methanol acidified with 0.1% formic acid (*v/v*) in the ultrasonic bath (IS-20, Intersonic, Olsztyn, Poland) twice for 30 min. The extracts were prepared in a solvent-to-solid ratio 1:20 (*v:w*) according to the procedure performed in our previous studies [47]. The content of compounds was expressed in micrograms per g of dry weight [μg/g d.w.].

### 4.2. Phytochemical Analysis

The isoquinoline alkaloids and phenolic compounds identification and quantification of *C. solida* and *P. lutea* were performed using liquid chromatography equipped with electrospray ionization-tandem mass spectrometry with a triple quadrupole analyzer.

Reference substances such as protopine, berberine, sanguinarine, chelidonine, chelerythrine were purchased from Extrasynthese (Genay, France); allocryptopine, coptisine, malic acid, *t*-aconitic acid, quinic acid, caffeic acid, chlorogenic acid, p-coumaric acid, vanillin, rutin, and quercetin were purchased from Sigma-Aldrich (St. Louis, MO, USA).

#### 4.2.1. Liquid Chromatography Mass Spectrometry

The analyzes were conducted using a Shimadzu Prominence UFLC system (Shimadzu, Kyoto, Japan). LC system was equipped with a binary solvent manager—LC-30 ADXR; a degasser—DGU-20A3; a column oven—CTO-10ASVP; an autosampler—SIL 20AXR; a system controller—CBM-20A. For compound separation, Kinetex column C18, 2.6 μm particle size, 100 × 3.0 mm (Phenomenex, Torrance, CA, USA) was used at a flow rate of 0.40 mL min^−1^. The mobile phase consisted of a mixture A–B composed of 10mM ammonium formate in water (A) and 0.1% formic acid in methanol (B). The methanol percentage was changed linearly as follows: 0 min, 10%; 10 min, 85%; 13 min, 85%; 16 min, 10%. Sample volume injection was 10 mL. Tandem mass spectrometer—LCMS-8030 (Shimadzu, Kyoto, Japan)—with a triple quadrupole mass spectrometer equipped with ESI source cooperating in both positive and negative ionization modes was used. LabSolution Ver. 5.6 (Shimadzu, Kyoto, Japan) software was used for quantitative data processing.

#### 4.2.2. Identification and Quantification

The multiple reaction monitoring (MRM) mode was used for identification and quantification of alkaloids and the remaining compounds. The identification was based on the retention time compared with the corresponding standards together with the ion intensity ratio of the chosen parent ion (Q), product ion (q) and previously identified compounds reported in the literature [48]. The limit of detection (LOD) was calculated according to a signal-to-noise ratio (S/N) of 3 and the limit of quantitation (LOQ) to S/N ratio of 10. The linearity of the method was studied for all of the chosen compounds based on five concentration points assessed in triplicate. The square correlation coefficient (r2) ≥ 0.99 was achieved for most of the compounds, or was very close. Quantitation was based on external standardization.

### 4.3. Experimental Design for Bioactivity Assays

In order to determine the antimicrobial activity of the tested substances, MIC (Minimum Inhibitory Concentration) assessment was conducted in 96-well titration micro-plates. Three reference strains were analyzed: *Staphylococcus aureus* ATCC 6538, *Pseudomonas aeruginosa* ATCC 15442, *Candida albicans* ATCC 1032 (obtained from ATCC, Manasas, VA, USA). Initially, the 0.5 McFarland (MF) density of the tested strain’s suspension in Tryptic Soya Broth (TSB) medium was prepared and diluted to 10^5^ Colony-Forming Units (CFU)/mL. Next, 100 μL of TSB medium was poured into the wells of the plate. Subsequently, 100 μL of the methanolic extract was added to the first well. Then, 100 μL of this solution (consisting of a mixture of methanolic extract and TSB) was transferred to the next well of 96-well plate. This operation was repeated 9 times. Finally, every of 10 wells of 96-well plate in a row, was filled with 100 μL of a solution containing decreasing concentrations of analyzed extracts. Next, 100 μL of bacterial/fungal suspension (105 cfu/mL) was added to each well. The final volume of 200 µL was obtained for each well. The plate was incubated at 37 °C and shaken (400 rpm/min.) for 24 h. Control of microorganisms’ growth (the culture without any of plant extracts) and control of sterility (medium only) were also performed. Moreover, antimicrobial activity of 100% methanol, 0.1% solution of formic acid, mixture of 100% methanol and 0.1% formic acid (4:0.5 ratio) and Octenisept (Schülke, Norderstedt, Germany)—clinically used antiseptic product of confirmed antimicrobial activity was examined as the positive control. After incubation, 20 μL of 1% solution of triphenyl tetrazolium chloride, TTC (Sigma-Aldrich, München, Germany) was introduced to the wells and incubated for 2 h/37 °C. A change of TTC to red formazan indicated the presence of metabolically active microorganisms. The MIC value was determined as the first colourless well, next to the red well.

### 4.4. Statistical Evaluation

Presented data of alkaloids and phenolic compounds content are mean values from 6 independent extractions ± standard deviation (SD). Statistical significance of the quantitative differences between extracts was estimated by one-way ANOVA with nonparametric Mann–Whitney *U* test at significance level *p* ≤ 0.05. All analyses were conducted using Statistica 13.1PL (StatSoft, Krakow, Poland, 2016).

## 5. Conclusions

The above data indicate the existence of much controversy regarding the types and number of alkaloids present in *C. solida* and *P. lutea*. It should be noted that there are relatively few studies on the phytochemical characterization of both species. Many of them were published in the seventies and nineties of the last century. Since then, analytical techniques have undergone enormous progress, leading to reduced measurement times and increased precision. Based on this, to make a broad picture of the phytochemical profiles of plants belonging to *Corydalis* genus, it would be necessary to examine the aerial and underground parts of multiple *Corydalis* species and related taxa, collected from different locations and different vegetation periods. Well-characterized plant material can stand a chance of applying it against clinical strains of pathogenic bacteria and fungi.

## Figures and Tables

**Table 1:** The content (µg/g d.w. ± SD) of quantitated compounds in aerial parts of *C. solida* and *P. lutea.*

No	Compound	Parent Ion (*m/z*)	Product Ion (*m/z*)	Ion Mode	Content Mean ± SD	
	ALKALOIDS				*C. solida*	*P. lutea*
1	protopine derivative	354	320, 260, 196	*+*	p	p
2	allocryptopine	369	352, 188, 290	*+*	328 ± 13.99 *	LOD
3	coptisine	320	292, 204, 262	*+*	154 ± 7.42 *	1526 ± 24.12
4	berberine	336	320, 292, 321	*+*	128 ± 6.79 *	197 ± 12.10
5	chelidonine derivative	370	356, 339	*+*	p	nd
6	chelidonine	354	275, 189, 247	*+*	58 ± 3.67 *	3 ± 0.69
7	chelerythrine	348	332, 304, 333	*+*	18 ± 1.31 *	4 ± 0.26
8	tetrahydroberberine	340	176, 149	*+*	p	p
9	tetrahydrocoptisine	324	176, 149	*+*	p	p
10	coptisine derivative	324	190	*+*	p	p
11	sanguinarine	332	274, 317, 246	*+*	35 ± 2.78 *	12 ± 0.89
12	protopine	320	303, 107, 124	*+*	440 ± 16.10 *	1036 ± 30.62
Other Compounds						
13	malic acid	133	115, 71	*-*	LOQ	LOQ
14	*trans*-aconitic acid	173	85, 129	*-*	LOQ	LOQ
15	quinic acid	191	85, 93	*-*	LOQ	LOQ
16	*trans*-caffeic acid	179	135, 134, 89	*-*	21 ± 1.52 *	32 ± 4.90
17	chlorogenic acid	353	191, 85, 93	*-*	1 ± 0.13 *	32 ± 1.51
18	*p*-coumaric acid	163	119, 93, 117	*-*	28 ± 1.71 *	16 ± 1.85
19	vanillin	151	136, 92, 108	*-*	11 ± 0.93	13 ± 1.69
20	quercetin	301	151, 65, 121	*-*	177 ± 9.67 *	3247 ± 66.43
21	rutin	609	300	-	LOQ	LOQ

p—present, identification was based on mass spectra with no reference substances; nd—not detected; LOD—limit of detection; LOQ—limit of quantification; means marked with an asterisk (*) within lines differ at significance level *p* ≤ 0.05 in a Mann–Whitney *U* test.

**Table 2:** The content (µg/g d.w. ± SD) of quantitated compounds in underground parts of *C. solida* and *P. lutea*.

No	Compound	Parent Ion (*m/z*)	Product Ion (*m/z*)	Ion Mode	Content Mean ± SD	
	ALKALOIDS				*C. solida*	*P. lutea*
1	protopine derivative	354	320, 260, 196	*+*	p	p
2	allocryptopine	369	352, 188, 290	*+*	516 ± 21.52 *	6 ± 1.37
3	coptisine	320	292, 204, 262	*+*	233 ± 5.13 *	307 ± 17.36
4	berberine	336	320, 292, 321	*+*	78 ± 3.58 *	326 ± 8.40
5	chelidonine derivative	370	356, 339	*+*	p	nd
6	chelidonine	354	275, 189, 247	*+*	1 ± 0.05 *	5 ± 0.20
7	chelerythrine	348	332, 304, 333	*+*	7 ± 0.37 *	6 ± 0.12
8	tetrahydroberberine	340	176, 149	*+*	p	p
9	tetrahydrocoptisine	324	176, 149	*+*	p	p
10	coptisine derivative	324	190	*+*	p	p
11	sanguinarine	332	274, 317, 246	*+*	8 ± 0.28 *	36 ± 3.53
12	protopine	320	303, 107, 124	*+*	1125 ± 32.63 *	1934 ± 25.98
Other Compounds						
13	malic acid	133	115, 71	*-*	LOQ	LOQ
14	*trans*-aconitic acid	173	85, 129	*-*	LOQ	LOQ
15	quinic acid	191	85, 93	*-*	LOQ	LOQ
16	*trans*-caffeic acid	179	135, 134, 89	*-*	nd	nd
17	chlorogenic acid	353	191, 85, 93	*-*	6 ± 1.26 *	nd
18	*p*-coumaric acid	163	119, 93, 117	*-*	LOD	nd
19	vanillin	151	136, 92, 108	*-*	nd	nd
20	quercetin	301	151, 65, 121	*-*	19 ± 2.88 *	76 ± 4.64
21	rutin	609	300	-	nd	nd

p—present, identification was based on mass spectra with no reference substances; nd—not detected; LOD—limit of detection; LOQ—limit of quantification; means marked with an asterisk (*) within lines differ at significance level *p* ≤ 0.05 in a Mann–Whitney *U* test.

**Table 3:** Minimum Inhibitory Concentration (MIC) [mg/L] of water–methanolic extracts from *S. solida* and *P. lutea* herb and underground parts.

Plant Material	*S. aureus*	*P. aeruginosa*	*C. albicans*
*C. solida* herb	1.56	0.39	0.39
*C. solida* corms	1.56	0.39	0.39
*P. lutea* herb	0.39	0.39	0.39
*P.lutea* roots	1.56	0.39	0.39
Octenisept (method suitability control)	0.0001	0.00152	0.0001
